# Rensch’s rule in avian lice: contradictory allometric trends for sexual size dimorphism

**DOI:** 10.1038/s41598-019-44370-5

**Published:** 2019-05-27

**Authors:** Imre Sándor Piross, Andrea Harnos, Lajos Rózsa

**Affiliations:** 1 Department of Biomathematics and Informatics, University of Veterinary Medicine, Budapest, Hungary; 2 Balaton Limnological Institute, MTA Centre for Ecological Research, Tihany, Hungary; 3 Evolutionary Systems Research Group, MTA Centre for Ecological Research, Tihany, Hungary; 4 MTA-ELTE-MTM Ecology Research Group, Budapest, Hungary

**Keywords:** Coevolution, Sexual selection

## Abstract

Rensch’s rule (RR) postulates that in comparisons across closely related species, male body size relative to female size increases with the average size of the species. This holds true in several vertebrate and also in certain free-living invertebrate taxa. Here, we document the validity of RR in avian lice using three families (Philopteridae, Menoponidae, and Ricinidae). Using published data on the body length of 989 louse species, subspecies, or distinct intraspecific lineages, we applied phylogenetic reduced major axis regression to analyse the body size of females vs. males while accounting for phylogenetic non-independence. Our results indicate that philopterid and menoponid lice follow RR, while ricinids exhibit the opposite pattern. In the case of philopterids and menoponids, we argue that larger-bodied bird species tend to host lice that are both larger in size and more abundant. Thus, sexual selection acting on males makes them relatively larger, and this is stronger than fecundity selection acting on females. Ricinids exhibit converse RR, likely because fecundity selection is stronger in their case.

## Introduction

**Figure 1:**
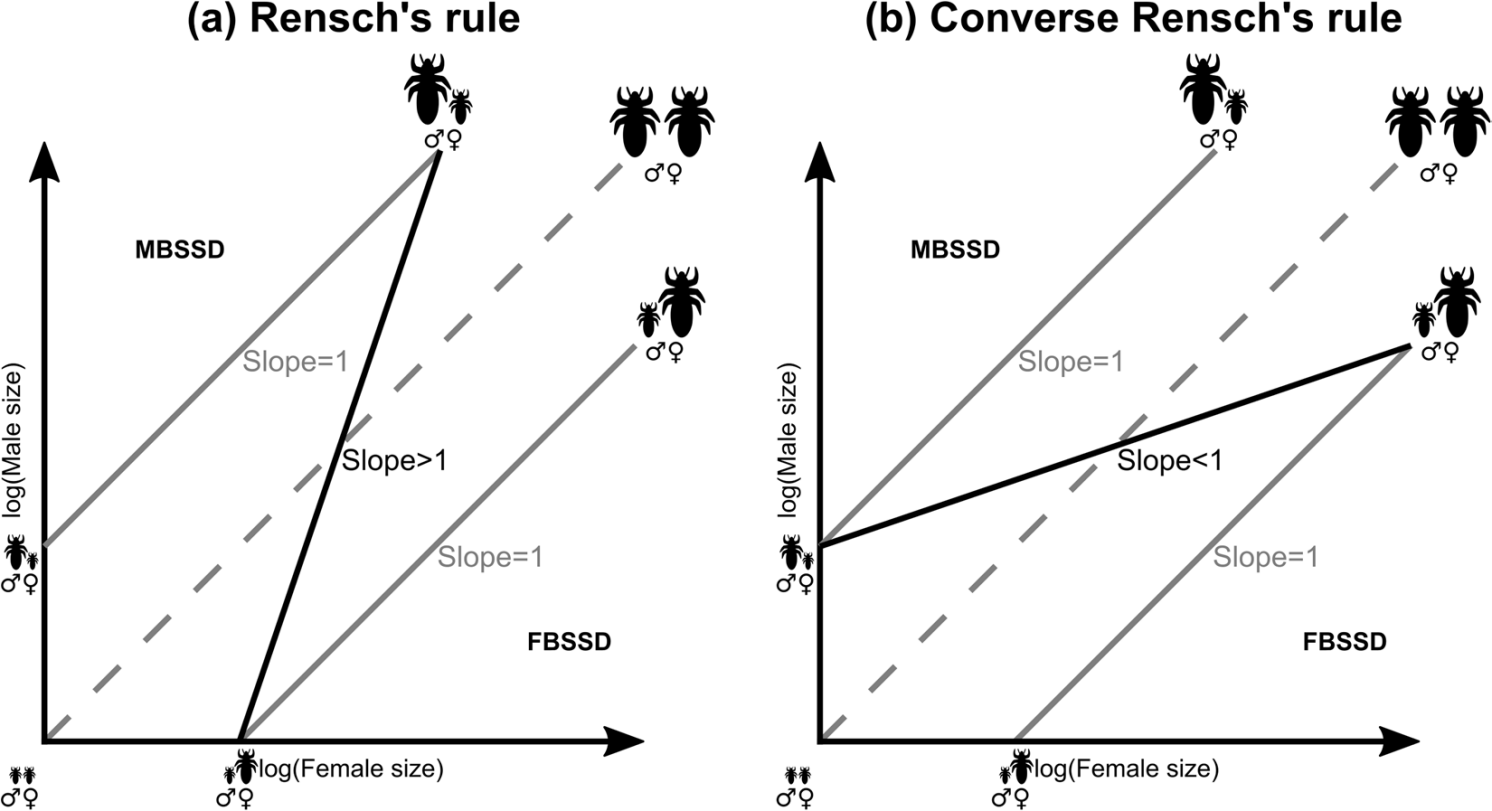
Graphical representation of Rensch’s rule and converse Rensch’s rule. When the logarithm of male body size is plotted against the logarithm of female body size, species with equal male and female sizes (no sexual size dimorphism: No SSD) are located along a line with a slope of 1 going through the origin (grey dashed line). Species deviating from it show SSD proportional to the distance from this line. Species where the males are larger (male-biased sexual size dimorphism: MBSSD) are located above, and species where females are larger (female-biased sexual size dimorphism: FBSSD) located below it. The slopes of the trend lines indicate whether the relative male size changes with the average size of the species. If relative male size does not change with the average size, the trend has a slope of 1 (grey solid lines). If relative male size increases with the average size, the trend has a slope > 1 (a, black solid line). This is called Rensch’s rule (**a**). Among species where the females are smaller (FBSSD), SSD decreases. When the males’ size exceeds the females’ size (MBSSD), the SSD increases. If relative male size decreases with the average size, the trend has a slope < 1 (b, black solid line). This is called converse Rensch’s rule (**b**). In this case, males are getting proportionally smaller with the average size of the species, meaning that SSD decreases in MBSSD species and increases in FBSSD species with size.

Body size is a fundamental trait of living organisms which influences most aspects of their biology. In sexually reproducing species, body size often differs between sexes. This is referred to as sexual size dimorphism (SSD). Male-biased sexual size dimorphism (MBSSD) refers to taxa the males of which are larger than the females, and female-biased sexual size dimorphism (FBSSD) refers to taxa the females of which are larger. When examining patterns of SSD among closely related animal species, Rensch^1^ observed that the relative male size (as compared to female size) increases with the average size of the species. In cases of taxa characterized by MBSSD, SSD increases as a consequence of the increasing relative male size. In taxa which exhibit FBSSD, the difference between the sexes diminishes with the increasing size of the species. This phenomenon is known as Rensch’s rule (RR). RR can be neatly visualised by plotting the male against the female sizes of different species on a logarithmic scale^2^. On the resulting graph, a group of species with a constant relative male size is positioned along trend lines of slope 1. When RR applies, the trend can be characterised by a line with a slope >1, meaning that relative male size increases with the female absolute size. See Fig. 1a for further details. The reversed relationship between relative male size and the size of the species is called Converse Rensch’s rule (CRR). In this case, relative male size decreases with the average size of the species, resulting in a decreasing SSD among MBSSD species and increasing SSD among FBSSD species. This defines a line with a slope <1 on the same graph. See Fig. 1b for further details.

Although there have been many studies focusing on RR, there is no general consensus about the causes for the allometry for SSD across closely related species. A thorough review by Fairbairn^3^ gathered a variety of functional hypotheses to explain RR. Dale *et al*.^4^ organised the existing explanations into three groups. We adopt this categorisation with the difference that we treat the fecundity selection hypothesis separately from the natural selection hypothesis. Note that the following hypotheses are not necessarily exclusive.

First, the evolutionary constraints hypothesis^3^ posits that the two sexes react to a different extent to the same selection pressures on body size. One cause of this may be that one sex has more additive genetic variance on body size, allowing it to respond more rapidly to selection. If the selection pressure varies among species, this can give rise to a pattern consistent with RR when males can respond more strongly and to CRR when females do.

Second, the natural selection hypothesis^3^ predicts that if the increase in the species’ body size reduces interspecific competition, then it enhances intersexual resource competition, resulting in a niche divergence between the sexes, and this niche divergence finds manifestation in divergences in body size.

Third, the sexual selection hypothesis^2,3^ assumes correlation — but not a total correspondence — between the body sizes of the sexes. When sexual selection on body size is stronger in one sex than the other, the other sex follows the growth of the first with more sexual selection pressure on it, but it cannot quite keep up, since there is only a correlation between the body sizes of the sexes. If sexual selection acts more heavily on males, RR is expected to turn up, regardless of whether the selection is for larger or smaller body size. When the males are selected to be larger, the female body size does not change as rapidly, resulting in RR. Stronger sexual selection on female body size results in CRR.

The “Fecundity selection” hypothesis^5^ claims that variation in SSD among species could be caused by a variation in the intensity of fecundity selection acting on female size. In this case, it is hypothesised that males show only a correlated response to changes in female body size. This process results in the emergence of a CRR pattern.

RR seems to hold in many taxa, primarily (but not exclusively) among vertebrates^2,6–8^. Dale *et al*.^4^ showed that polygynous bird species follow RR, while in cases of species with reversed sex-roles, the allometry follows CRR. Székely *et al*.^8^ argued that selection favours larger males in birds, where a larger size is advantageous in competition for females, while FBSSD develops in bird species where females compete with one another for males.

However, there are controversies concerning the applicability of the rule. The evidence for RR in FBSSD taxa is particularly scarce^9^. Blanckenhorn *et al*.^10^ reviewed the validity of RR in insects. Investigating data from seven insect orders, they found that RR applies to only half of the insect orders and, thus, may not be the norm in insects. CRR also occurs in insects^11,12^.

While parasitism is one of the most common life strategies on earth^13^, only a handful of papers have investigated RR among parasites. For instance, Poulin determined that RR applies to parasitic copepods^14^, but found no evidence for it among parasitic Nematodes^15^. Recently, Surkova *et al*.^16^ found RR among fleas, but not among parasitic mites.

Since parasitic lice (Insecta: Phthiraptera) reproduce sexually and exhibit remarkable sexually selected traits, including size dimorphism^17^, they constitute a suitable taxon to investigate the applicability of RR in parasites. Lice are obligate ectoparasites which complete their entire life cycle in the host plumage or pelage^18^. Two suborders of lice are found on avian hosts: Amblycera and Ischnocera. Philopteridae, the only avian lice in the latter suborder, are particularly specialised to move on feathers and hide in plumage^19^. Birds mainly counter philopterids by preening.

Menoponidae is the largest family in the suborder Amblycera. Menoponids are less specialized, and they can be found on any body parts of the host, although their oviposition and feeding are more restricted to certain areas. They live on the skin, in the fluffy underlayer of the plumage, and also on feather shafts^20–22^. They are more agile than ischnocerans, and they use their mobility to escape from preening^18^. A few genera are more specialized, for example *Actornithophilus* and *Colpocephalum* species can live inside feather shafts, and *Piagetiella* species can live inside the pouch of pelicans^23^.

Ricinid lice — also from the suborder Amblycera — are mostly restricted to small-bodied passerines and hummingbirds (Trochilidae), with a few species parasitizing medium-sized passerines like thrushes (*Turdus* spp.) and orioles (*Oriolus* spp.). They tend to be relatively large-bodied compared to their hosts^24^, and the prevalence and intensity of their infestations tend to be low^25^.

As in most other sexually reproducing animals, the males constitute the more competitive sex among lice and, thus, their body size may be more influenced by intrasexual rivalry. Mating time in louse species can range from 10–15 seconds up to 40 hours, although the latter can be interpreted as mate guarding behaviour by the males. The males of several philopterid lice possess modified antennae, which they use to grasp the female’s thorax during copulation to ensure attachment to the female. This is important, as it prevents rivals from dislodging them during copulation^17,19^. Another form of male-male competition among these creatures is sperm competition, which is the most widespread form of sexual competition in arthropods^26^. Larger males can produce greater quantities of sperm, and, therefore, they are more competitive in this context^17,27,28^.

Generally, females are the larger sex in lice^18^. Harnos *et al*.^24^ showed that females of the Philopteridae, Menoponidae, and Ricinidae families follow Harrison’s rule (HR)^29^. This rule postulates that larger hosts tend to harbour larger parasites. In the case of females of the philopterid lice *Columbicola columbae*, fertility is positively related to body size^30^. The authors proposed a microevolutionary mechanism to explain the emergence of HR. When *C. columbae* find themselves on relatively smaller hosts, host defences (preening) select them for smaller sizes better able to fit in the interbarb spaces. On relatively larger hosts, fecundity selection selects for larger females.

The purpose of our present study is to test whether RR applies to avian lice. Since the epidemiological and morphological characteristics of different louse taxa exhibit markedly different relationships to host characters^17,24^, first, we investigate three major families of avian lice; the ischnoceran family Philopteridae and the amblyceran families Menoponidae and Ricinidae. Harnos *et al*.^24^ also compared host-parasite body size allometries across the four philopterids guilds (called ‘wing lice’, ‘body lice’, ‘head lice’, and ‘generalists’) formerly outlined by Johnson *et al*.^31^ Since this categorization is challenged by recent studies on the *Brueelia*-complex, where a species-level categorization is required^32,33^, we prefer to discontinue comparisons between ‘ecomorph’ categories until a widely accepted new categorization will be published. In the second part of our study, we analyse RR in menoponid and philopterid lice separately from three different host orders. In the hope of gaining more insight into the underlying mechanism behind RR in avian lice, we also provide descriptive statistics linking SSD and the body size of lice to the body mass of their hosts.

## Methods

### Data collection

Data were obtained from species descriptions and are identical with the dataset recently used by Harnos *et al*.^24^, although that study used only female total body length values. Most of the body length data refer to species, however, when available, data regarding distinct subspecies or distinct populations associated with different host species were included as separate louse lineages. In cases of multiple measurements of the same parasite species (or subspecies, or lineage) from different sources in the literature, we averaged the values. Louse body size was expressed as total body length of slide-mounted specimens. Slide-mounting is a well standardized method for preserving and measuring lice^34^, thus its potential distorting effects are expected to be similar across samples. Research efforts may differ across host taxa, potentially introducing a certain degree of bias in our data set. Table 1 and Table 2 contain the sample sizes.

**Table 1:** Means and standard deviations of male and female body lengths, relative male sizes (male body length/female body length), and host weights (g) for each investigated group, n is the number of operational taxonomic units (species, populations, or host specific lineages).

		Male body length (μm)		Female body length (μm)		Male body length/Female body length (μm)		Host mass (g)		n
		Mean	s.d.	Mean	s.d.	Mean	s.d.	Mean	s.d.	
Louse families	Philopteridae	2063	939	2385	919	0.85	0.08	1213	5422	514
	Menoponidae	1764	685	2081	688	0.84	0.1	846	1656	375
	Ricinidae	2999	570	3686	747	0.82	0.06	28	23	100
Philopterid and menoponid lice from different host orders	Philopteridae from Passeriformes	1526	246	1843	255	0.83	0.07	113	148	90
	Menoponidae from Passeriformes	1383	275	1693	300	0.82	0.06	124	211	97
	Philopteridae from Charadriiformes	1673	199	2018	213	0.83	0.04	248	236	90
	Menoponidae from Charadriiformes	1643	330	2037	366	0.81	0.09	262	278	56
	Philopteridae from Galliformes	2229	679	2534	715	0.88	0.09	1051	989	97
	Menoponidae from Galliformes	1788	296	1965	237	0.91	0.12	1189	1000	34

**Table 2:** Louse species (or subspecies, or lineages) closest to the 2.5%, 50% (median) and 97.5% quantiles of relative male size (male body length/female body length), with the species’ relative male size, male body length (µm), female body length (µm), host species and the host’s weight (g).

		Quantile	Male body length/ Female body length (μm)	Male body length (μm)	Female body length (μm)	Host weight (g)	Louse species name	Host species name
Louse families	Philopteridae	2.5%	0.71	1322	1873	66	*Brueelia mahrastran*	*Turdoides striata*
		50%	0.85	1560	1830	74	*Brueelia straminea*	*Dendrocopos major*
		97.5%	1.03	2800	2720	217	*Strongylocotes subconiceps*	*Crypturellus soui*
	Menoponidae	2.5%	0.67	1550	2330	291	*Hohorstiella gigantea*	*Columba oenas*
		50%	0.84	1800	2150	634	*Colpocephalum leptopygos*	*Plegadis falcinellus*
		97.5%	1.08	2710	2500	2419	*Holomenopon goliath*	*Anseranas semipalmata*
	Ricinidae	2.5%	0.72	3400	4700	68	*Ricinus elongatus*	*Turdus philomelos*
		50%	0.81	3180	3920	16	*Ricinus serratus*	*Serinus flaviventris*
		97.5%	0.92	2930	3190	12	*Ricinus dendroicae*	*Dendroica striata*
Philopterid and menoponid lice from different host orders	Philopteridae from Passeriformes	2.5%	0.70	1411	2012	61	*Brueelia magnini*	*Turdoides fulva*
		50%	0.83	1429	1716	70	*Brueelia addoloratoi*	*Turdus rufiventris*
		97.5%	0.98	2460	2500	570	*Philopterus ocellatus*	*Corvus corone*
	Menoponidae from Passeriformes	2.5%	0.70	1190	1700	18	*Menacanthus eurysternus*	*Tichodroma muraria*
		50%	0.82	1600	1960	200	*Myrsidea bakeri*	*Corvus kubaryi*
		97.5%	0.91	1730	1900	294	*Colpocephalum fregili*	*Corvus splendens*
	Philopteridae from Charadriiformes	2.5%	0.74	1680	2280	192	*Saemundssonia (Saemundssonia) africana*	*Vanellus albiceps*
		50%	0.83	1630	1960	96	*Saemundssonia (Saemundssonia) platygaster theresae*	*Jacana spinosa*
		97.5%	0.92	1520	1660	61	*Saemundssonia (Saemundssonia) chathamensis*	*Thinornis novaeseelandiae*
	Menoponidae from Charadriiformes	2.5%	0.66	1150	1750	655	*Austromenopon atrofulvum*	*Sterna caspia*
		50%	0.82	1690	2050	53	*Actornithophilus ceruleus*	*Procelsterna cerulea*
		97.5%	0.97	1650	1700	136	*Actornithophilus pediculiodes*	*Arenaria interpres*
	Philopteridae from Galliformes	2.5%	0.71	1875	2640	1135	*Lipeurus maculosus*	*Phasianus colchicus*
		50%	0.87	2000	2290	749	*Lipeurus sarissa*	*Rhizothera longirostris*
		97.5%	1.05	2770	2650	1330	*Lipeurus raymondi*	*Acryllium vulturinum*
	Menoponidae from Galliformes	2.5%	0.75	1556	2070	504	*Menacanthus lyali*	*Alectoris chukar*
		50%	0.90	1680	1870	1490	*Amyrsidea (Cracimenopon) jacquacu*	*Penelope jacquacu*
		97.5%	1.19	2090	1750	379	*Menacanthus werneri*	*Polyplectron napoleonis*

To analyse RR separately for different host orders, information on host taxonomy was obtained from IOC World Bird List v 8.2^35^. The vast majority of ricinid lice in our dataset are from passeriform birds (97 out of 106 records), therefore, this louse family was excluded. In the cases of philopterids and menoponids, we chose the three most common host orders of the two families. The dataset with references to sources are available in the Supporting Information as comma separated value files (see Supplementary Data S1).

### Louse phylogeny

The molecular phylogeny of lice is poorly understood; therefore, we adapted the louse tree of Harnos *et al*.^24^ without any further modifications. This tree is basically a compilation based on published taxonomies^25,36–45^ and interpreted as an approximation of the true phylogeny of avian lice. The phylogeny of lice (see Supplementary Data S2) in CAIC format is available in the Supporting Information.

### Statistical analyses

We fitted phylogenetic reduced major axis regression^46,47^ (pRMA) for log-transformed male vs. female body lengths separately for the three louse families, and for philopterids and menoponids from three different host orders. Deviation from isometry was accepted when the slope of the fitted line significantly (P value ≤ 0.05) differed from 1. We also estimated phylogenetic signal expressed as Pagel’s λ^48^. All analyses were carried out in R 3.4.3^49^. We used a jackknife method to investigate the influence of each observation on the slopes of the fitted lines. We refitted all pRMA models by leaving out each observation one at a time, and we recorded the results for each model, calculated the difference in the slope estimates, and observed if the significance of its deviation from isometry changed. We applied the ape 5.0 package^50^ to import and handle phylogenetic trees, the phytools 0.6–44 package^51^ to fit pRMAs, the RcmdrMisc 1.0–5 package^52^ to calculate descriptive statistics, and the ggplot2 2.2.1 package^53^ to create a visual rending of the data. The R code we used is available in the Supporting Information (see Supplementary Code S3).

## Results

### Descriptive statistics

The means and standard deviations of male and female body lengths, the relative male sizes (expressed as the ratio of male to female body length), host body masses, and the sample sizes are reported in Table 1.

As mentioned above, our sample may be biased, i.e. it may not necessarily represent the true distribution of lice across host body size classes. In this sample, the mean host mass is the largest among the philopterids, with a considerable standard deviation. Host masses tend to be lower for menoponid lice, though they still cover a wide range. As expected, ricinids were found only on small-bodied birds. The means of relative male sizes are similar between louse families. Menoponids have a somewhat shorter body length than philopterids, while ricinids are the largest among the three families.

In our sample, the differences between menoponids and philopterids grouped by three different host orders suggest that larger-bodied bird orders (Passeriformes < Charadriiformes < Galliformes) harbour lice with larger mean male and female body length and also a slightly larger mean relative male length. The mean host masses of philopterids and menoponids marginally differ in the three orders, with menoponids found on slightly larger hosts.

Table 2 shows the louse species (or subspecies, or lineage) closest to the 2.5%, 50% (median), and 97.5% quantiles of relative male size (ratio of male to female body length) for each group investigated. Relative male size, male and female body lengths, and host size and species are also reported. On a family level, philopterid and menoponid lice seem to show a pattern consistent with RR; with the increase of female body length, the male body lengths increase faster (thus the relative male size increases), with increasing host body weights. Ricinids, on the other hand, seem to exhibit a CRR pattern. Relative male size decreases as the female size increases, and host body masses also decrease with the increase of relative male size. Grouped by host orders, philopterids and menoponids from Passeriformes also show a trend consistent with RR, while in other groups, these descriptive statistics do not clearly match either RR or CRR.

### Results of pRMA models

**Figure 2:**
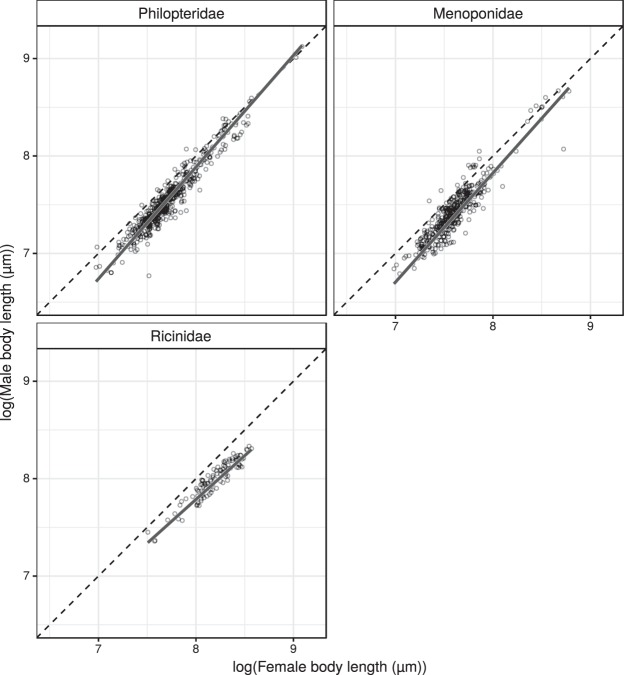
Allometric relationships of the louse families. Allometric relationship between log-transformed male and female body lengths (µm) with isometric slopes (dashed lines) and fitted phylogenetic reduced major axis regression lines (solid lines) by louse families.

**Figure 3:**
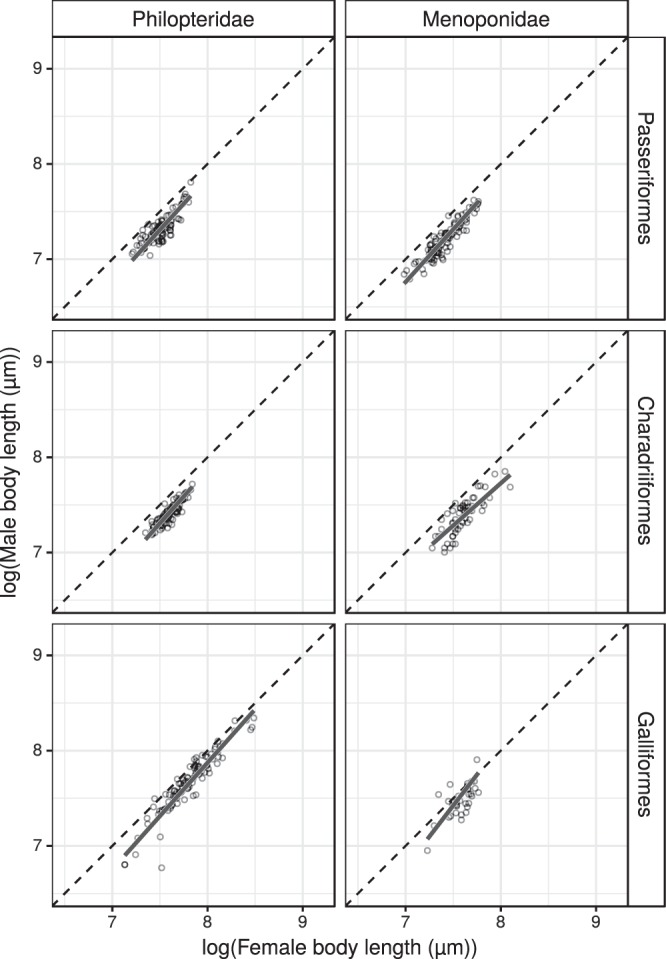
Allometric relationships of philopterid and menoponid lice from passeriform, charadriiform, and galliform birds. Allometric relationship between log-transformed male and female body lengths (µm) with isometric slopes (dashed lines) and fitted phylogenetic reduced major axis regression lines (solid lines) by louse families and host orders.

Results of the pRMA regressions, the estimated phylogenetic signals, and sample sizes are reported in Table 3. For visual representations of the data and the fitted lines, see Fig. 2 for families, Fig. 3 for philopterids and menoponids from different host orders. On a family level, both philopterid and menoponid lice show male-female allometric relationships consistent with RR. Ricinid lice exhibit a CRR trend (allometric slope < 1), which is surprising. Grouping the lice by host orders, we observed that RR applies to menoponids from Passeriformes and philopterids from both Charadriiformes and Galliformes. In all cases in which allometries consistent with RR were confirmed, the estimated slopes have numerically similar values, ranging from 1.11 to 1.16.

**Table 3:** Results of the phylogenetic reduced major axis regressions of log (male body length (µm)) on log (female body length (µm)) for the three louse families, and for philopterids and menoponids from three different host orders. The estimated phylogenetic signals (λ) and sample sizes (n, number of operational taxonomic units: species, populations, or host specific lineages) are also reported.

		Intercept	Slope	R^2^	t-value	degrees of freedom	P value (H_0_: true slope = 1)	Phylogenetic signal (λ)	Effect of the most influential point on the slope	n
Louse families	Philopteridae	−1.29	**1.15**	0.86	8.21	360.47	**<0.0001**	0.91	0.46%	514
	Menoponidae	−1.14	**1.12**	0.76	4.47	272.57	**<0.0001**	0.93	1.51%	375
	Ricinidae	0.60	**0.90**	0.88	3.07	70.01	**0.0030**	0.86	0.46%	100
Philopterid and menoponid lice from different host orders	Philopteridae from Passeriformes	−0.94	1.10	0.71	1.67	66.86	0.1004	0.01	1.30%	90
	Menoponidae from Passeriformes	−0.98	**1.11**	0.86	2.62	68.39	**0.0107**	0.56	2.58%	97
	Philopteridae from Charadriiformes	−1.14	**1.13**	0.76	2.29	65.7	**0.0255**	0.67	2.77%	90
	Menoponidae from Charadriiformes	0.62	0.89	0.64	1.45	42.88	0.1537	0.50	7.07%	56
	Philopteridae from Galliformes	−1.10	**1.12**	0.87	3.05	68.29	**0.0033**	0.73	1.39%	97
	Menoponidae from Galliformes	−2.33	*1.30*	0.44	1.99	28.19	*0.0565*	0.90	12.06%	34

### Jackknife diagnostics of pRMA models

In each pRMA model where deviation from isometry was confirmed, leaving out any single observation (data point) from the regression model changed neither the significance of the results nor the general direction of the slope (whether it is smaller or larger than 1). The effects of the most influential points on the slope — expressed as a percentage of the slope estimate — are reported in Table 3. In the cases of these models, the maximal effects range from 0.46% to 3.09%.

In some models where the deviation from isometry was not confirmed, certain observations can have a notable influence on the estimated slope. In these cases, leaving out particular observations from the model can result in a significant (P value ≤ 0.05) deviation from isometry. In the regression model of menoponids from Galliformes 9 out of 34 have this property.

## Discussion

We have shown that two major taxa of avian ectoparasites, philopterid and menoponid lice, clearly obey RR. Host order level analysis in these families also confirmed RR separately in the case of Menoponidae from Passeriformes and Philopteridae from Charadriiformes and Galliformes. In contrast, however, ricinids follow CRR, where males get relatively smaller (as compared to females) with increasing size of the species.

Deviance from isometry was not proven in some cases. Philopterids from Passeriformes show a numerically similar allometric trend compared to menoponids from the same host order. The model explains less variance in the data among philopterids from these birds, perhaps indicating weaker mechanisms behind RR than among menoponids. Although not significant, the steep slope of menoponids from Galliformes and the slope of Menoponidae from Charadriiformes, which are consistent with CRR, are interesting trends. They indicate that accounting for host taxonomy and life history traits is a promising direction in investigating the underlying causes for RR.

Although without formal comparison, in all cases where RR was supported, the allometric slopes showed similar values. Based on this, it would be hard to come up with different interpretations of the results. Philopterids altogether show a somewhat steeper, but generally similar allometric trend compared to menoponids.

The non-exclusive alternative hypotheses explaining RR and Converse RR may more or less apply to our findings. Lice can respond quickly to selection pressures on body size^30^, but the genetics of their body size in relation to sex is not known. Furthermore, we lack knowledge about possible niche divergences between the sexes.

Sexual selection is known to be an influential agent of evolution in several taxa of parasites^54,55^, including parasitic lice^17,27,28^. Given that larger-bodied host species tend to have more prevalent and more abundant infestations of menoponid and philopterid lice^56–58^, we expect that the males in these populations tend to coexist with more rivals and also face an increased level of outbreeding due to a higher chance of multiple infections. This strengthens intrasexual competition^59^, and it also probably exerts a selection pressure favouring larger males. Our descriptive statistics in Table 2 empirically support this view; in many cases (namely in the Philopteridae and Menoponidae families together and separately from the Passeriformes), relative male size tends to increase with host body size. This probably indicates that in menoponids and philopterids, sexual selection due to male-male rivalry exerts stronger selection pressure on male size than fecundity selection exerts on female size. Contrarily, CRR observed in ricinid lice may indicate that fecundity selection is stronger on female body size than sexual selection is on male body size.

Based on our findings, it appears that similar selection pressures shape the evolution of SSD across avian lice, except for the family of Ricinidae.

## Supplementary information

Supplementary Data S1: Louse family data

Supplementary Data S2: Louse family phylogeny

Supplementary Code S3: Analysis in R

## Data Availability

All data analysed in the study are available in the Supporting Information.
